# Biogenic Silver Nanoparticles: Assessment of Their Cytotoxicity, Genotoxicity and Study of Capping Proteins

**DOI:** 10.3390/molecules25133022

**Published:** 2020-07-02

**Authors:** Magdalena Wypij, Tomasz Jędrzejewski, Maciej Ostrowski, Joanna Trzcińska, Mahendra Rai, Patrycja Golińska

**Affiliations:** 1 Department of Microbiology, Nicolaus Copernicus University, Lwowska 1, 87100 Torun, Poland; 279419@stud.umk.pl; 2 Department of Immunology, Nicolaus Copernicus University, Lwowska 1, 87100 Torun, Poland; tomaszj@umk.pl; 3 Department of Biochemistry, Nicolaus Copernicus University, Lwowska 1, 87100 Torun, Poland; maciejost@umk.pl; 4 Nanobiotechnology Lab., Department of Biotechnology, SGB Amravati University, Amravati, Maharashtra 444602, India; mahendrarai@sgbau.ac.in

**Keywords:** biosynthesis, capping proteins, cytotoxicity, genotoxicity, silver nanoparticles

## Abstract

The development of nanotechnology in the last two decades has led to the use of silver nanoparticles (AgNPs) in various biomedical applications, including antimicrobial, anti-inflammatory, and anticancer therapies. However, the potential of the medical application of AgNPs depends on the safety of their use. In this work, we assessed the in vitro cytotoxicity and genotoxicity of silver nanoparticles and identified biomolecules covering AgNPs synthesized from actinobacterial strain SH11. The cytotoxicity of AgNPs against MCF-7 human breast cancer cell line and murine macrophage cell line RAW 264.7 was studied by MTT assay, cell LDH (lactate dehydrogenase) release, and the measurement of ROS (reactive oxygen species) level while genotoxicity in *Salmonella typhimurium* cells was testing using the Ames test. The in vitro analysis showed that the tested nanoparticles demonstrated dose-dependent cytotoxicity against RAW 264.6 macrophages and MCF-7 breast cancer cells. Moreover, biosynthesized AgNPs did not show a mutagenic effect of *S. typhimurium*. The analyses and identification of biomolecules present on the surface of silver nanoparticles showed that they were associated with proteins. The SDS-PAGE (sodium dodecyl sulfate–polyacrylamide gel electrophoresis) analysis revealed the presence of 34 and 43 kDa protein bands. The identification of proteins performed by using LC-MS/MS (liquid chromatography with tandem mass spectrometry) demonstrated their highest homology to bacterial porins. Capping biomolecules of natural origin may be involved in the synthesis process of AgNPs or may be responsible for their stabilization. Moreover, the presence of natural proteins on the surface of bionanoparticles eliminates the postproduction steps of capping which is necessary for chemical synthesis to obtain the stable nanostructures required for application in medicine.

## 1. Introduction

Nanotechnology and related nanomaterials have become the most leading and promising areas for scientific research and technological development. The creation and utilization of nanomaterials and nanodevices already have multiple applications in various fields [1]. However, the greatest expectations are for its application in medicine, with the direct impact this could have on the quality of health in future societies. Nanomedicine brings nanotechnology and medicine together, in order to develop novel diagnostics, therapies and improve existing treatments [2]. Although recent advances in nanotechnology have broadened the potential applications for silver nanoparticles (AgNPs), at the same time increasing human exposure to these materials and raising anxiety regarding their adverse biological effects [3].

The synthesis of nanoparticles (NPs) may be performed by chemical, physical, and biological approaches. However, the use of various chemical and physical processes generates the consumption of a high amount of energy as well as toxic and hazardous chemicals [4]. Alternatively, the green synthesis of nanoparticles using bacteria, actinomycetes, fungi, yeasts, algae and plants is a natural, simple, and ecofriendly method [5]. Moreover, the biological synthesis results in high yield, good solubility, high stability and biocompatibility of obtained nanoparticles [6]. In the extracellular synthesis of silver nanoparticles, the reducing agents, including amino acids, membrane proteins, NAD(P)^+^ reductases, dehydrogenases and various secondary metabolites, are involved, whereas the nanoparticle capping agents are formed by extracellular proteins, enzymes or peptides [7,8].

Out of all nanomaterials, silver nanoparticles are the most frequently biosynthesized nanostructures [9]. They have a surface/volume ratio much greater than the corresponding bulk material which increases their bioactivity, e.g., against pathogens or cancer cells, and makes them good carriers for other molecules (drugs, proteins, probes) attached to their surface [5,10]. Overall, bionanoparticles are considered as more stable and biocompatible due to the covering by microbial-derived capping agents [11]. The association of biomolecules with silver nanoparticles has been shown to modify the stability of the nanoparticles, as well as their behavior in the physiological environment [12]. Interestingly, these proteins have unique functionalities and a defined primary structure that enables various possibilities for the surface modifications and attachment of other compounds such as drugs and therapeutics. These complex biomolecule-nanoparticle conjugates may be utilized for various medical applications [4,5,13]. Moreover, Poblete and co-authors [12] suggested that interaction between proteins and silver nanoparticles play a pivotal role in the biocompatibility of nanoparticles, and ultimately, its antimicrobial performance. Unfortunately, the mechanisms that underlie such stabilization, as well as the exact role of various biomolecules and amino acid moieties, remain unknown [12]. Studies on the identification of molecules covering biosilver nanoparticles also remain in their infancy and nothing is known about identification of capping agent molecules of AgNPs synthesized from actinobacteria.

This work is a continuation of our comprehensive studies of silver nanoparticles synthesized by actinobacterial strain SH11, which was isolated from pine forest soil and identified as *Streptomyces kasugaensis* M338-M1^T^ and *S*. *celluloflavus* NRRL B-2493^T^ (99.8% similarity, both) [14]. Biosynthesized silver nanoparticles were previously characterized in terms of physico-chemical properties and assessed for their antimicrobial activity against Gram-positive and Gram-negative bacteria, alone and in combination with commercially used antibiotics. They showed antibacterial activity separately, but importantly, they enhanced the antibacterial activity of selected antibiotics when used in combination. Moreover, the TEM (transmission electron microscope), zeta potential and FTIR (Fourier transform infrared spectroscopy) analysis confirmed the formation of small in size, polydispersed, stable, and capped with biomolecules AgNPs, respectively [14].

The present study was designed to assess the cytotoxicity of biosynthesized AgNPs from actinobacterial strain SH11 towards MCF-7 human breast cancer cell line and murine macrophage cell line RAW 264.7 by MTT (3-(4,5-dimethylthiazol-2-yl)-2,5-diphenyltetrazolium bromide) assay, the measurement of LDH (lactate dehydrogenase) release and cellular ROS (reactive oxygen species) level, as well as their genotoxicity using the bacterial reverse mutation assay (Ames test). Moreover, the first-time identification of proteins involved in the formation and stabilization of AgNPs synthesized from actinobacterial strain SH11 was performed.

## 2. Results

### 2.1. Characterization of AgNPs

**Figure 1:**
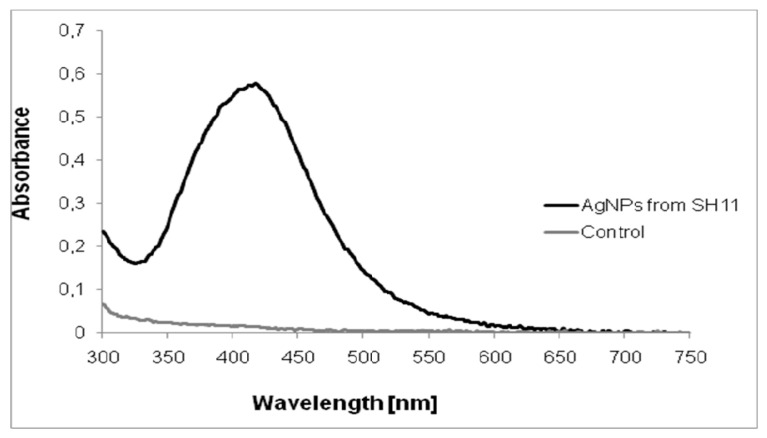
UV–Visible spectrum of silver nanoparticles synthesized from actinobacterial strain SH11.

The ultraviolet-visible (UV–vis) spectroscopy analysis revealed a sharp narrow peak with a maximum absorbance at *λ* = 419 nm (Figure 1), which is in the range specified for silver nanoparticles and confirmed their presence in the reaction mixture.

**Figure 2:**
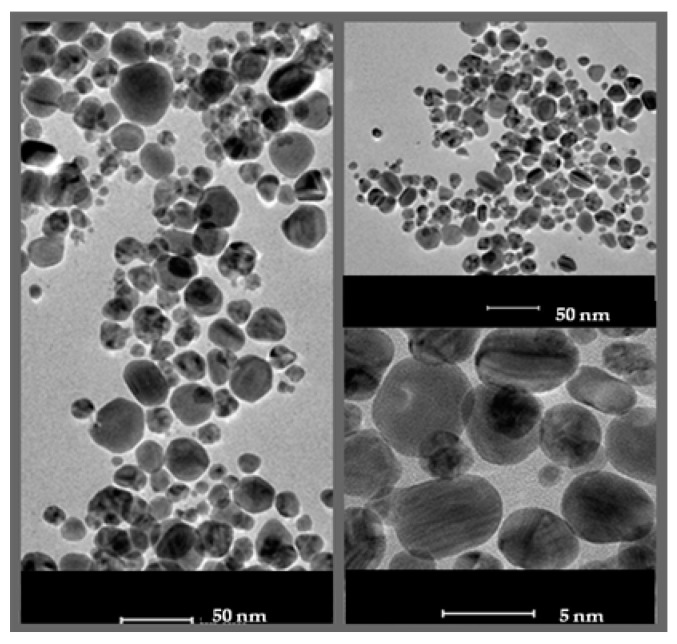
Transmission electron micrograph of silver nanoparticles synthesized from actinobacterial strain SH11.

TEM analysis confirmed that the AgNPs were polydispersed and spherical with a size range of 4–32 nm and a mean size of 15.9 nm (Figure 2).

**Figure 3:**
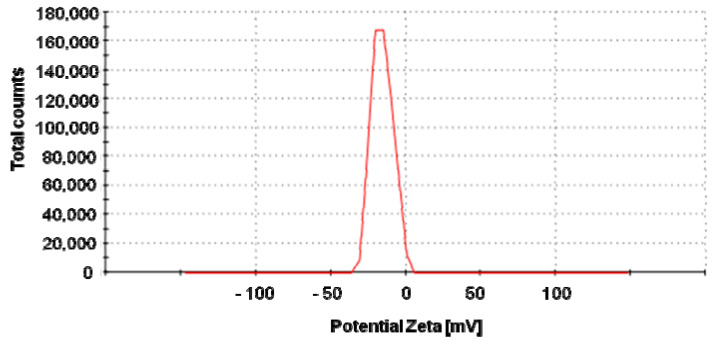
Zeta potential graph of the silver nanoparticles (AgNPs) synthesized from SH11 strain (−17.1 mV).

Biosynthesized nanoparticles were negatively charged (−17.1 mV) with good stability (Figure 3).

### 2.2. Cytotoxicity of AgNPs against RAW 264.7 Macrophages and MCF-7 Cancer Cells

**Figure 4:**
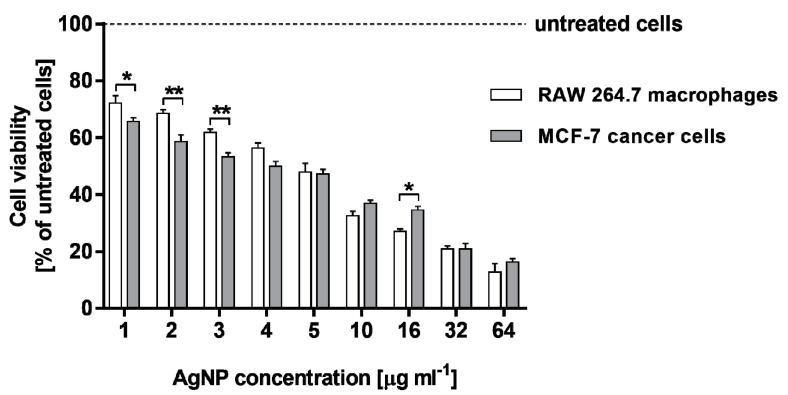
Cytotoxic activity of AgNPs from actinobacterial strain SH11 against MCF-7 human breast cancer cell line and murine macrophage cell line RAW 264.7 estimated by MTT assay. Asterisks indicate significant differences between the viability of RAW 264.7 macrophages and MCF-7 cancer cells (** *p* < 0.01; * *p* < 0.05).

The cytotoxic effect of AgNPs on the murine RAW 264.7 macrophages and human MCF-7 breast cancer cells was evaluated using two different assays. The cell viability was detected by MTT test, whereas the level of cell death was determined by measuring lactate dehydrogenase (LDH) leakage. Both the cell lines were stimulated with AgNPs in the concentration range of 1–64 µg·mL^−1^. Both RAW 264.7 and MCF-7 cell viabilities were significantly and similarly decreased in response to AgNP stimulation in a dose-dependent manner compared to untreated cells (*p* < 0.001 for all the tested concentrations of AgNPs), as can be seen in Figure 4. The viability of RAW 264.7 cells was found to be from 72.1 ± 2.7% to 12.7 ± 3.0%, whereas that measured for MCF-7 cells from 65.6 ± 1.4% to 16.2 ± 1.3% for AgNP concentration range from 1 to 64 µg·mL^−1^, respectively.

**Figure 5:**
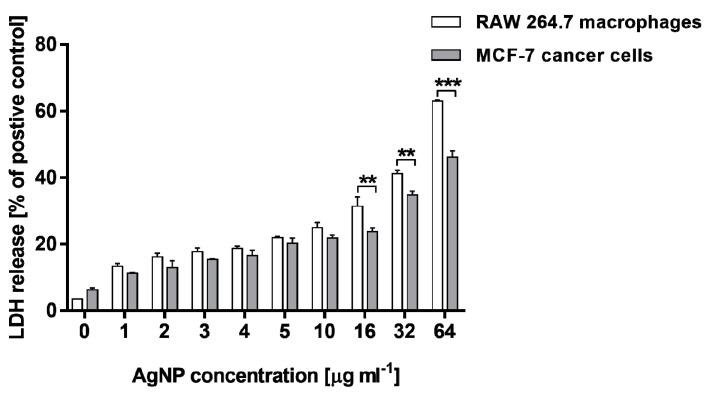
Lactate dehydrogenase (LDH) release from MCF-7 human breast cancer cells and murine macrophage cells RAW 264.7 after treatment with various concentrations of biosynthesized AgNPs. Asterisks indicate significant differences between the levels of LDH released from RAW 264.7 macrophages in comparison with MCF-7 cells (*** *p* < 0.001; ** *p* < 0.01).

The LDH release assay supported the results of the MTT test. As compared with the positive control, cells treated with 0.8% Triton X-100 solution for 45 min and AgNP-stimulated RAW 264.7 macrophages, as well as MCF-7 cells, exhibited an increase in LDH leakage in a dose-dependent manner. The cytotoxicity measured for RAW 264.7 cells was from 13.3 ± 0.9% to 62.9 ± 0.3%, whereas that estimated for MCF-7 was in a range of 11.2 ± 0.2%–46.1 ± 2.0%. Moreover, RAW 264.7 cells treated with the highest concentrations of AgNPs (16, 32 and 64 µg·mL^−1^) released greater amounts of LDH in comparison with MCF-7 cells (Figure 5). The amounts of LDH measured in culture media from untreated control cells were 3.5 ± 0.4% and 6.2 ± 0.6% for RAW 264.7 and MCF-7 cells, respectively. All the tested AgNP concentrations increased LDH leakage compared with untreated cells (*p* < 0.001).

Using data from MTT and LDH assays, the half inhibitory concentration (IC_50_), which quantifies the concentration of each compound to inhibit cell growth by half, was obtained (Table 1). IC_50_ is the concentration of the compound that causes 50% of the maximum effect, thus a 50% reduction of viable cells (MTT assay) or a 50% release of LDH (LDH assay). The values of IC_50_ obtained by the LDH assay were found to be higher than those obtained by MTT test. However, a comparison of the all IC_50_ results for both cell lines indicated that treatment of RAW 264.7 macrophages with the tested AgNPs resulted in the higher cytotoxic effect in terms of metabolic activity and membrane integrity than the stimulation of MCF-7 cancer cells, which can be clearly observed, especially for the LDH assay (112.9 ± 0.08 µg·mL^−1^ vs. 43.5 ± 0.08 µg·mL^−1^).

### 2.3. Estimation of Reactive Oxygen Species Levels in RAW 264.7 Macrophages and MCF-7 Cancer Cells

**Figure 6:**
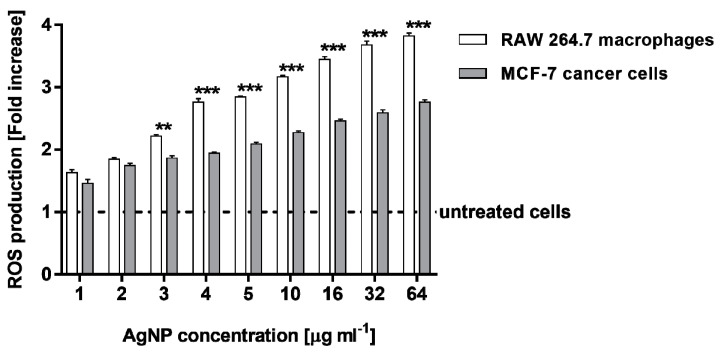
ROS (reactive oxygen species) production level in MCF-7 human breast cancer cells and murine macrophage cells RAW 264.7, after treatment with various concentrations of biosynthesized AgNPs. Asterisks indicate significant differences between the ROS production in RAW 264.7 macrophages in comparison with MCF-7 cancer cells (*** *p* < 0.001; ** *p* < 0.01).

To assess the capacity of AgNPs to generate intracellular reactive oxygen species (ROS), RAW 264.7 macrophages and MCF-7 cancer cells were stimulated with different concentrations of AgNPs for 24 h and stained with 2′,7′-dichlorofluorescein diacetate (DCFH-DA). Biosynthesized AgNPs significantly increased ROS generation in both the cell lines in a dose-dependent manner (Figure 6). All the tested AgNP concentrations increased ROS production compared with untreated cells (*p* < 0.001). Importantly, RAW 264.7 macrophages were more sensitive to AgNPs and released a greater amount of ROS when they were stimulated with AgNPs in a concentration range of 3–64 µg·mL^−1^(from 2.21 ± 0.03 to 3.82 ± 0.05-fold increase, respectively), in comparison with MCF-7 cancer cells (a fold increase in the range of 1.86 ± 0.04–2.76 ± 0.04).

### 2.4. Ames Test

The study of mutagenicity showed that biosynthesized AgNPs in tested concentrations (0.15–100 µg per plate) did not cause a reversal of mutation in *S. typhimurium* cells when compared to positive control (2-nitrofluorene). However, the inhibitory effects of biosynthesized AgNPs against bacterial cells were observed. The number of colonies decreased in a concentration of AgNPs equal to 6 µg·mL^−1^ or higher ones (Table 2). The increased number of revertant colonies found in the positive control indicated that the test system responded appropriately.

### 2.5. Analysis of AgNP Capping Proteins

**Figure 7:**
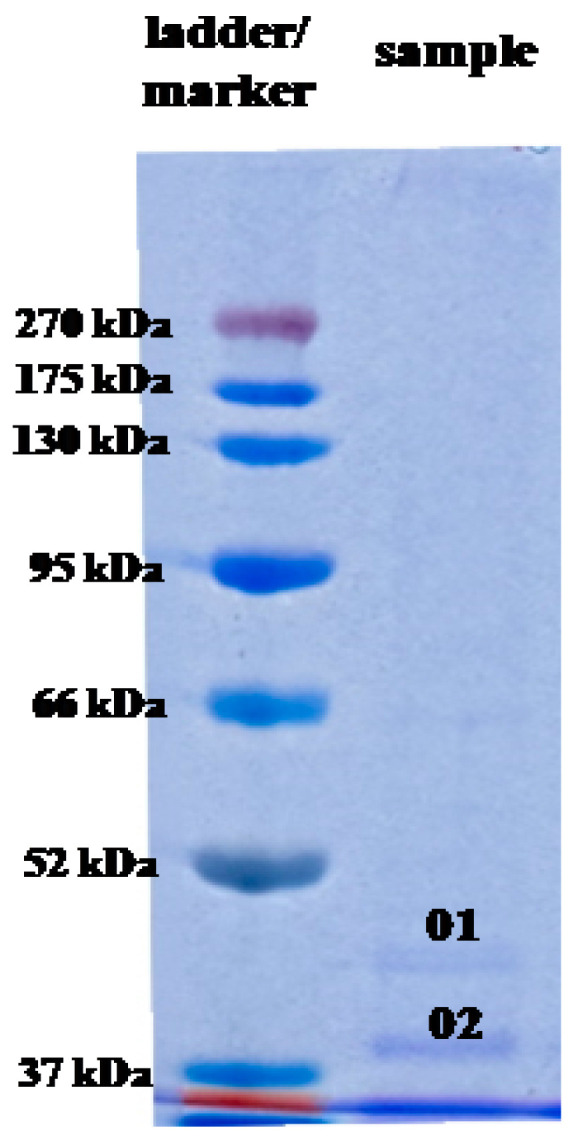
SDS-PAGE of proteins associated with AgNPs biosynthesized from actinobacterial strain SH11.

In this study, SDS-PAGE separation of the cell-free supernatant treated with AgNO_3_ revealed the presence of two protein bands which were absent in the control sample (cell-free supernatant without incubation with Ag^+^ ions) (Figure 7).

These protein bands, labeled as 01 and 02, exhibited molecular mass, corresponding to 43 and 34 kDa, respectively. These bands were cut out from the gel, subjected to trypsin digestion and analyzed by LC-MS/MS. The obtained tryptic peptides were identified based on *m*/*z* value, as shown in Table 3. Both excised bands were identified as peptides with the highest homology to proteins from OprD family porin found in *Pseudomonas fluorescens* (pI = 5.73) and porin from *Cupriavidus* sp. HMR1 (pI = 9.1), respectively.

## 3. Discussion

It is well known that the size, as well as the tendency of the agglomeration and dissolution of nanomaterial, determine their toxic effect on biological systems [15]. Size is an important feature of nanomaterials, as it affects their cellular uptake, physical properties, and interactions with biomolecules. The smaller the size of nanoparticles, the easier their penetration through biological membranes [16], and the higher surface area of nanomaterials, thus their surface activity [15]. It was reported that the nanomaterials of diameter under 12 nm can easily pass through the blood-brain barrier [17,18,19], and that the smaller nanoparticles cause the dose-dependent increase in oxidation and DNA damage [20]. In the present study, we showed that actinobacterial strain SH11 synthesized small (mean size 15.9 nm) and spherical silver nanoparticles. These results are in line with our findings on this strain reported previously by Wypij et al. [14]. Biosynthesized nanoparticles showed morphology and size similar to those synthesized from other actinobacterial strains [21,22,23,24]. The results of zeta potential measurements (−17.1 mV) indicated good stability of the synthesized nanoparticles due to the electrostatic repulsion between particles in the solution. It has been claimed that the closer the zeta potential value to −30 mV, the more stable are metal nanoparticles, thus their tendency to form aggregates is lower as their harmful effect to biological systems [15,25,26]. Negatively charged nanoparticles synthesized by actinomycetes were previously reported by many authors [14,21,23,24]. Rathod et al. [21] and Wypij et al. [23,24] showed that AgNPs synthesized by filamentous actinobacterial strains had zeta potential in a range from −14.7 to −18.0 mV, thus similar to that found in AgNPs from strain SH11. However, it was suggested that differences in the zeta potential of biogenic nanoparticles might be related to different conditions of the synthesis process and differences between bacterial isolates [26,27,28].

As the silver nanoparticles have been increasingly recognized for their important industrial applications in many fields, such as medicine, food, and agriculture, the human exposure to these nanoparticles is inevitable [29]. For example, for biomedical purposes, especially in in vivo applications, the toxicity of AgNPs is a critical factor to consider when evaluating their potential [30]. Therefore, nanotoxicology research is now gaining attention. Although many studies on the toxicity of AgNPs in a variety of cancerous and non-cancerous cells have been reported [23,24,31,32], the mechanisms underlying the toxicity are still far from clear.

There are a number of reports showing that the cytotoxic and genotoxic effect of AgNPs is dependent on their ability to penetrate inside the cells via diffusion (translocation), endocytosis or phagocytosis [33], thus their size, as mentioned previously. It is believed that the AgNPs themselves or by Ag^+^ ion release after penetration into cells can stimulate the production of radical oxygen species (ROS), resulting in oxidative stress [34,35,36,37,38,39,40]. Generally, at low levels, ROS regulate various cellular functions, but at higher levels, ROS apart from the damaging effects to the cell membrane through the release of lactate dehydrogenase [41], cellular proteins, lipids and DNA, triggers the cell to respond by activating pro-inflammatory signaling cascades, and ultimately induces programmed cell death by either apoptosis or necrosis [37,42,43,44,45]. The induced apoptosis in a variety of human cancers, including breast cancer, ovarian cancer, and lung cancer after the increasing production of ROS, has been reported previously [32,46,47]. Moreover, the cellular uptake of AgNPs also leads to their accumulation in the mitochondria, thereby inducing mitochondrial dysfunction, such as a reduction in the mitochondrial membrane potential and promoting ROS creation. As a consequence, intracellular proteins and nucleic acids are damaged [48]. The disruption of the mitochondrial respiratory chain induced by AgNPs also interrupts ATP synthesis, thereby resulting in DNA damage [49,50]. AgNPs can also interact with the membrane proteins and activate signalling pathways, leading to the inhibition of cell proliferation [41]. Finally, Ag^+^ ions released from AgNPs initiate cascades or a series of events that lead to intracellular toxicity, termed as the “lysosome-enhanced Trojan horse effect” [36,51].

The analysis of IC_50_ values clearly indicates that the stimulation of RAW 264.7 macrophages with AgNPs resulted in being more cytotoxic, especially in relation to membrane integrity, than the treatment of MCF-7 cancer cells.

Our results showed that the tested AgNPs significantly increased ROS production in both the cell lines, however, this effect was higher in the macrophages. Therefore, we presume that a higher sensitivity of the macrophages to the AgNPs may be a ROS-dependent phenomenon. Moreover, the study on various cell lines such as macrophages (RAW 264.7, J774.1), epithelial cells (A549, A498 HepG2) and neurons (Neuro 2A) showed that the former exhibited the highest sensitivity to AgNP stimulation. The explanation of this phenomenon may be related to the scavenger receptor pathway and the scavenger function of macrophages that increase their sensitivity to the effects of nanoparticles [52,53]. This increased cytotoxicity of AgNPs may be attributed in part to its internalization by macrophages, followed by a subsequent Ag^+^ ion release in the lysosome compartments. Numerous reports indicate that not only ROS production, but also the generation of silver ions, is responsible for the toxic effects of AgNPs [35,36,38,39]. However, Ag^+^ ions released from AgNPs can also induce ROS generation [40], especially for cellular uptake through endocytosis [51]. Finally, 6phagocytosis of AgNPs by macrophages can generate ROS that stimulate the production of tumor necrosis factor α (TNF-α). The increase of TNF-α causes the damage of cell membrane and apoptosis. Thus, it is speculated to be caused by the ionization of AgNPs in the cell, which is expressed as a Trojan-horse type mechanism [36].

Similarly, the cytotoxic effect of AgNPs derived from actinobacterial strain OF1 on non-cancerous (3T3 fibroblasts) and cancerous (HeLA) cells (IC_50_ value 4 and 3.8 µg·mL^−1^, respectively) was found in our previously reported studies when a MTT assay was performed [23]. However, another silver nanoparticles synthesized from two actinobacterial strains IF11 and IF17 revealed lower cytotoxicity toward the above cell lines recorded as 8.3 and 28.5 µg·mL^−1^ (AgNPs from strain IF11) and 28.3 and 58.3 µg·mL^−1^ (AgNPs from strain IF17) [24]. Therefore, it can be concluded that actinobacterial-mediated silver nanoparticles exhibit variable cytotoxic effects against mammalian cells, which can be dependent on physico-chemical properties of biosynthesized nanoparticles. Moreover, the high cytotoxicity of AgNPs from strain SH11 against cancer cells qualify them as a potential targeted anticancer agent for biomedical applications, in the future.

In this work, the Ames test (OECD standard assay) was used for the screening of biosynthesized AgNPs as a potential mutagen and for identifying potential human carcinogens [54,55]. This test is commonly used by the pharmaceutical industry for the evaluation of different drugs and chemicals before using them for clinical trials [56]. In our research, no increase in the number of revertant bacterial colonies on plates treated with silver nanoparticles compared to untreated and positive control plates indicated a lack of mutagenic potential of the AgNPs synthesized by actinobacterial strain SH11. However, decreasing the number of colonies after treatment with higher concentrations of AgNPs confirms the inhibitory effect of AgNPs on bacterial growth. Negative results of the genotoxicity of AgNPs by the Ames test were also reported by other authors [57,58,59]. They suggested that AgNPs did not induce the large-scale DNA damage which could be detected by the Ames test [57]. It should also be considered that AgNPs themselves have antimicrobial activity and their toxicity to the bacterial strains may reduce the sensitivity of the test [54,60].

As mentioned previously, different kinds of biomolecules, such as enzymes, proteins and biosurfactants present in microorganisms, may be involved in the biological synthesis of NPs as a reducing agent [61]. The proteins of bacterial origin are also considered as a capping agent for nanoparticles [7,8,61]. The mycogenic nanoparticles have already demonstrated the greater stability of silver nanoparticles due to the capping of fungal proteins. The nanoparticles synthesized by chemical and physical means need additional capping agents, while the biogenic nanoparticles are capped by the proteins present in extracts used for synthesis [62]. The capping agents present in biogenic nanoparticles protect Ag^0^ from oxidation to silver ions. The protein-based capping is beneficial for the attachment of biomolecules/drugs or genetic material for delivery to the human cells [63]. In another report, the role of extracellular proteins secreted by an endophytic *Aspergillus tubingensis* in capping and stabilization of silver nanoparticles, and the supramolecular interactions between silver nanoparticles and proteins was explained [64]. The authors identified the protein responsible for capping which was covalently attached to silver nanoparticles primarily through S–Ag bonds due to cysteine residues (HS–) and with few N–Ag bonds from H_2_N–groups. Furthermore, they reported supramolecular interactions by electrostatic and additional protein-protein interactions, and found proteins including acid phosphatase (EC 3.1.3.2), serine carboxipeptidase (EC 3.4.21.26), glucoamylase (1,4-α-d-glucanglucohydrolase, EC 3.2.1.3) and glucanosyltransferase (EC 2.4). These proteins are responsible for the metabolic activities of *A. tubingensis* [64].

The silver nanoparticles synthesised by peel extract of *Citrus sinensis* were stabilized by germin-like protein showing oxalate oxidase activity, another protein with glutathione *S*-transferase activity, and the third protein was hypothesized as an aspartic-type endopeptidase structure [9]. The understanding of protein-nanoparticle interaction is essentially required to comprehend the activity of nanoparticles. After the adsorption of proteins on nanoparticles, the physicochemical properties, including surface charge, size and surface composition, change dramatically. The protein-nanoparticle complex regulates various biological activities such as cellular uptake, bioavailability, and toxicity, etc. Barbalinardo et al. [65] studied chemically synthesized nanoparticles capped with proteins present in biologial fluids, and suggested that such proteins affected interactions of the AgNPs with cells. Many of these proteins increase the internalization of AgNPs into cells by specific binding with their cognate receptors expressed on cell membranes. It was found that AgNPs were internalized via endocytosis [66]. Moreover, the results suggest that the cellular uptake is mediated by presence of the protein corona, and therefore, the inherent toxic activity of AgNPs is related to these proteins [65].

The binding of proteins on a nanoparticle depends on surface energy. High surface energy encourages the better binding of protein with nanoparticles [67]. Moreover, the protein-nanoparticles interaction and protein-protein interaction regulates the adsorption of proteins [67].

The structure of proteins and molecular weight do not influence much on the surface activities of proteins. Usually, the proteins contain hydrophobic patches on their surface that determine their interaction with nanoparticles. The isoelectric point (pI) of protein depends on the concentration at which the least SPR appears [68].

However, based on available reports, it can be stated that knowledge on bionanoparticle capping agents is still in its infancy, particularly on bacteria. There is no thorough research on the identification of biomolecules capping the silver nanoparticles synthesized by microorganisms. Therefore, in this work, the proteins associated with biosynthesized AgNPs were subjected to analyses. Two protein bands (34 and 43 kDa) showing the highest homology to proteins from porin family were recognized. It is well established that porins form membrane channels allowing the transport of molecules across lipid bilayer membranes and they have ability to translocate from the outer bacterial membrane into host cell membranes, where it modulates the infection process [69]. Translocated porin may induce apoptosis by causing a rapid calcium influx, followed by the activation of the calcium-dependent cysteine protease calpain, as well as proteases of the caspase family. This effect is observed in the cancer cells and monocytic cell lines [70]. Based on these findings, we suppose that the tested protein-capped AgNPs may have important implications for the observed cytotoxicity of AgNPs. Although other authors [11] found several protein bands associated silver nanoparticles synthesized using cell-free extract of phytopathogenic soil-borne fungus *Macrophomina phaseolina* (Tassi) Goid, which showed molecular weights between 50 and 116 kDa, these proteins were not identified by the authors.

Therefore, further studies are needed to understand the precise role of biomolecules associated with silver bionanoparticles.

## 4. Materials and Methods

### 4.1. Bionanoparticle Synthesis

The biogenic synthesis of AgNPs from actinobacterial strain SH11 was performed as previously described by Wypij et al. [14]. Briefly, strain grew in yeast extract-malt extract broth pH 5.5 [71], for 7 days at 27 °C. The bacterial biomass was then collected by centrifugation (6000× *g* for 10 min) and washed three times with sterile distilled water. Biomass was then re-suspended in sterile distilled water and incubated at 27 °C for 3 days for cell autolysis. The autolysate was centrifuged at 6000× *g* for 15 min and obtained supernatant combined with silver nitrate (AgNO_3_; final concentration 0.001 mol·L^−1^). The reaction mixture was incubated for 3 days at 25 °C in the darkness.

### 4.2. Characterization of Biosynthesized AgNPs

The formation of AgNPs was monitored visually by the color change of the reaction mixture from colorless to yellowish-brown and using ultraviolet (UV)-visible spectroscopy (Nano Drop ND2000, Thermo Scientific, Waltham, MA, USA), in a wavelength range from 200 to 800 nm, at a resolution of 1 nm.The supernatant, untreated with silver nitrate, was maintained as the control. Biosynthesized AgNPs were separated from the solution by centrifugation (13,000× *g* for 1 h) and dried at 40 °C for mass evaluation (mg).

Biosynthesized nanoparticles were routinely characterized for size, shape and stability.

#### 4.2.1. Transmission Electron Microscopy Analysis

The size and morphology of the AgNPs from actinobacterial strain SH11 were analyzed by using transmission electron microscopy(TEM) (FEI Tecnai F20 X-Twintool, Fei, Hillsboro, OR, USA), operatingat an acceleration voltage of 100 kV. The one drop of AgNP solution in molecular grade sterile water was deposited on a carbon-coated copper grid (400 μm mesh size). The samples were then dried at room temperature and analyzed. The obtained data were assessed by Statistica Software (StatSoft Inc., Tulsa, OK, USA).

#### 4.2.2. Zeta Potential Analysis

The surface charge of biosynthesized AgNPs, which affects their stability, was estimated in a colloidal suspension of AgNPs based on measurement of their zeta potential.The AgNP solution was diluted 10 times with water and sonicated at 20 Hz for 15 min (Sonic Ruptor 250, Omni Int., Kennesaw, GA, USA) to break down NP aggregates. The mixture was then filtered through a 0.22-μm Millipore filter and analyzed using Zetasizer (Malvern Instruments Ltd., Malvern, UK).

### 4.3. Cell Culture

A MCF-7 human breast cancer cell line (Lot. 13K023) was obtained from the European Collection of Authenticated Cell Cultures (ECACC; Salisbury UK). The cells were cultured at 37 °C in a humidified atmosphere of 5% CO_2_ in a complete RPMI 1640 medium containing 2 mM l-glutamine, heat-inactivated 10% fetal bovine serum (FBS), 100 µg·mL^−1^ streptomycin and 100 IU·mL^−1^ penicillin and non-essential amino acids (all compounds from Sigma-Aldrich, Darmstadt, Germany). MCF-7 cells were removed from the cell culture flask using 0.25% trypsin-EDTA solution (Sigma-Aldrich, Darmstadt, Germany).

Murine macrophage cell line RAW 264.7 (cat. no. 91062702) was obtained from the European Collection of Authenticated Cell Cultures (Salisbury, UK). The cells were cultured in Dulbecco’s modified Eagle’s medium (DMEM) supplemented with 10% FBS, 100 µg·mL^−1^ streptomycin and 100 IU·mL^−1^ penicillin (all compounds from Sigma-Aldrich). Macrophages were maintained at 37 °C in a 5% CO_2_/95% humidified atmosphere and subjected to no more than 15 cell passages. The cells were passaged using a cell scraper.

### 4.4. Cell Viability

For the determinationof the level of cell viability, the MTT assay was performed. This test detects the reduction of MTT (3-(4,5-dimethylthiazolyl)-2,5-diphenyl-tetrazolium bromide; Sigma Aldrich) by mitochondrial dehydrogenase to blue formazan product, which reflects the normal functioning of mitochondria, and hence, the metabolic rate of cells. For each experiment, 1 × 10^4^ MCF-7 cells, as well as RAW 264.7 macrophages, were seeded in 96-well tissue culture plates in 200 µL of suitable complete culture medium. The plates were incubated at 37 °C in 5% CO_2_ for 24 h to allow cell adhesion, prior to the silver nanoparticle testing. Then, the different concentration (1, 2, 3, 4, 5, 10, 16, 32 and 64 µg·mL^−1^) of AgNPs in the corresponding cell culture medium (RPMI 1640 medium for MCF-7 cells or DMEM medium for RAW 264.7 macrophages, respectively) was added to the culture. Blank wells (without cells) contained a specific concentration of AgNPs in the appropriate culture medium. The plates were incubated for 24 h under the same conditions. After treatment, the cells were washed with phosphate-buffered and 100 µL per well of MTT/culture medium (without phenol red) solution (5 mg·mL^−1^ of MTT reagent in PBS; RPMI 1640 medium for MCF-7 cells and DMEM medium for RAW 264.7 cells) was then added to each well. Culture plates were incubated at 37 °C for 4 h. Subsequently, the MTT/culture medium solution was removed, and the formazan product formed by viable cells was dissolved in 100% DMSO (100 µL per well). The plate was mixed horizontally for 10 min and the optical density was measured at 570 nm (with a reference wavelength of 630 nm) using a Synergy HT Multi-Mode Microplate Reader (BioTek Instruments, Winooski, VT, USA). The results were expressed as the percentage of untreated cells, which was served as 100%. The percentage of cell viability was computed according to the following formula:

% cell viability = ((At − Abt)/(Ac − Abc)) × 100(1)

where, At—absorbance value of tested compound; Abt—absorbance value of blank well contained culture medium with the corresponding compound concentration; Ac—absorbance value of control cells; Abc—absorbance value of blank well contained culture medium.

All data were from three independent experiments, with six wells for each experiment. The 50% inhibitory concentrations (IC_50_) were also determined using GraphPad Prism 7.0 (GraphPad Software Inc., La Jolla, CA, USA).

### 4.5. Cytotoxicity Assay

Cells were seeded in 96-well cell culture plates at a density of 1 × 10^4^ cells per well in 100 µL of a complete growth medium for 24 h at 37 °C, in a humidified incubator. The cells were then stimulated with various concentrations of AgNPs for 24 h. Culture cell supernatants (50 μL) were transferred into a new 96-well microplate and the cytotoxicity was assessed using CytoTox 96^®^ Non-Radioactive Cytotoxicity Assay (Promega Corporation, Madison, WI, USA), according to the manufacturer’s instructions. The CytoTox 96^®^ Assay quantitatively measures lactate dehydrogenase (LDH), a stable cytosolic enzyme that is released upon cell lysis. Released LDH in culture supernatants is measured with an enzymatic assay, which results in the conversion of a tetrazolium salt (iodonitrotetrazolium violet; INT) into a red formazan product. The amount of color formed is proportional to the number of lysed cells. The cells in the positive control wells were treated with 0.8% Triton X-100 solution (provided by the manufacturer) for 45 min. These cells released a maximum amount of LDH, which was served as 100%. In the negative control wells, the cells were incubated in culture media alone. Blank wells contained the corresponding concentration of AgNPs, or culture medium without cells. The amount of formazan which is proportional to the amount of LDH released from dead cells was measured spectrophotometrically at 490 nm (Synergy HT Multi-Mode Microplate Reader; BioTek Instruments, Winooski, VT, USA). The level of LDH released in the AgNP-treated cells was reported as a percentage of Triton X-100-stimulated cells. All calculations were performed after respective blank absorbance subtraction. All data were from three independent experiments with five wells for each experiment. The 50% inhibitory concentrations (IC_50_) were also determined using GraphPad Prism 7.0 (GraphPad Software Inc., La Jolla, CA, USA).

### 4.6. Measurement of Cellular Reactive Oxygen Species Levels

To evaluate the generation of reactive oxygen species (ROS) in MCF-7 and RAW 264.7 cells induced by AgNPs, ROS accumulation was detected using 2′,7′-dichlorodihydrofluoresceindiacetate DCFH-DA (Sigma-Aldrich, Darmstadt, Germany). DCFH-DA is a non-fluorescent compound which, upon being taken up by passive diffusion into cells, is hydrolyzed by esterases to yield non-permeable DCFH. In the presence of ROS, DCFH is oxidized to the fluorescent DCF. The cells at a density of 25 × 10^3^ cells per well were seeded in 96-well tissue culture plates in 200 µL of suitable complete culture medium and pre-incubated at 37 °C for 24 h. The cells were then stimulated with the different concentrations of AgNPs for the next 24 h. After treatment, the cells were washed twice with PBS and incubated with 20 µM DCFH-DA (200 µL per well) at 37 °C for 30 min in the dark. Subsequently, DCFH-DA solution was removed and 100 µL of PBS was added to each well. The fluorescence was measured using a Synergy HT Multi-Mode Microplate Reader (BioTek Instruments, Winooski, VT, USA), with excitation at 485 nm and emission at 528 nm. All data were from three independent experiments with five wells for each experiment. The results were expressed as the fold change relative to equivalent control untreated cells. The ROS level was computed according to a similar formula, as described for the MTT test, where the absorbance values were changed to the measured fluorescence values.

### 4.7. Statistical Analysis

All values are reported as means ± standard error of the means (SEM) and were analyzed using analysis of variance followed by the Bonferroni multiple comparisons test, with the level of significance set at *p* < 0.05. Statistical analyses were performed with GraphPad Prism 7.0 (La Jolla, CA, USA).

### 4.8. Ames Test

To assess the potential carcinogenic effect of AgNPs, the Ames test was performed. The potential reversal mutation was tested by using the *Salmonella typhimurium* TA98 strain, which is a mutant for the biosynthesis of histidine and unable to grow in medium without this amino acid. *S. typhimurium* test strain TA98 was obtained from Trinova Biochem GmbH Corporation (Giessen, Germany). The cells of *Salmonella typhimurium* (TA98) were treated with different concentrations of biosilver nanoparticles, namely 0.15, 0.25, 0.5, 1.0, 1.5, 3.0, 6.0, 12.5, 25.0, 37.5, 50.0 and 100.0 μg per plate. Briefly, the 100 μL of an overnight culture (1–2 × 10^8^ CFU·mL^−1^) of *S. typhimurium* (TA98) growing in Oxoid no. 2 nutrient broth at 37 °C, was treated with AgNPs at relevant concentration and incubated for 2 h at 37 °C, with shaking at 80 rpm [16]. The positive control was TA98 strain of *S. typhimurium* treated with 2-nitrofluorene (Sigma Aldrich, Darmstadt, Germany) at a concentration of 3 μg per plate. The negative control was bacterial inoculum non-treated with silver nanoparticles or 2-nitrofluorene. The samples, as well as positive and negative controls, were then diluted with 2 mL of molten top agar (Trinova Biochem, Germany) and poured on minimum salts agar (Trinova Biochem, Germany) in Petri plates. The plates were then incubated at 37 °C for 48 h, and observed for revertant colonies. The experiment was performed in triplicate for each treatment.

### 4.9. Analyses of Capping Proteins

#### 4.9.1. Preparation of Samples for Identification of Proteins

The bacterial pellet of SH11 strain was washed trice with sterile distilled water (see above) and suspended in 50 mL of a 50 mM Tris-HCl buffer (pH 8.0), containing 300 mM NaCl, 10% (*v*/*v*) glycerol and 1 mM EDTA. The mixture was sonicated (Omni Ruptor sonic homogenizer, Omni Int., Kennesaw, GA, USA) three times for 20 s, and centrifuged at 10,000 × *g* for 15 min. The cell-free supernatant was used for the biosynthesis of AgNPs by adding silver nitrate (final concentration 0.001 mol·L^−1^). The reaction mixture was incubated in the darkness for 72 h. The proteins of the cell-free supernatant without the incubation with Ag^+^ ions were also analyzed. This modified method of silver nanoparticle fabrication was performed to obtain more efficient bands on polyacrylamide gel. The total protein concentration of the samples was determined by the Bradford method [72].

#### 4.9.2. SDS-PAGE

The electrophoresis under denaturing conditions (SDS-PAGE) was performed using 10% (*w*/*v*) running gel and 4% (*w*/*v*) stacking gel, according to the method described by Ogita and Markert [73]. The samples containing 20 µg of protein were applied into the wells of the polyacrylamide gel. The separation was be carried out at 150 V, until the dye reached the end of the separating gel. After electrophoresis, the gel was stained using a Coomassie Brilliant Blue R-250 [74].

#### 4.9.3. Identification of Proteins by LC-MS/MS

The protein bands obtained after SDS-PAGE were cut out from the gel and subjected to LC-MS/MS analysis in the Laboratory of Mass Spectrometry, Institute of Biochemistry and Biophysics, Polish Academy of Sciences, Warsaw, Poland. The proteins were digested with trypsin and analyzed by nanoAcquity UPLC (Waters, Etten-Leur, Netherlands) coupled to an Orbitrap Velos mass spectrometer (Thermo Scientific, Waltham, MA, USA). LC-MS/MS data were processed using MASCOT program (http://www.matrixcience.com/).

## 5. Conclusions

This work, a continuation of our previous research on silver nanoparticles from the SH11 strain and their potential biomedical application, was designed to evaluate the in vitro cytotoxicity towards non-cancerous and cancerous cell lines and the genotoxicity in *S. typhimurium* cells. In addition, the proteins of AgNP capping agents were identified, which is a pioneer study. UV-vis, TEM and zeta potential analyses confirmed the synthesis of silver nanoparticles from actinobacterial strain SH11, which were characterized with small size and good stability. These properties of AgNPs may affect their easier penetration into cells. Moreover, biogenic nanoparticles were capped with biomolecules of natural (bacterial) origin. The identification of capping proteins showed highest homology to proteins from porin family found in other bacteria. The presence of capping proteins may affect the cellular uptake, bioavailability, functionality and toxicity of AgNPs, which are important aspects in their use as therapeutic agents, especially in targeted therapies. Although analyses showed that the biosynthesized AgNPs demonstrated dose-dependent cytotoxicity in both tested cell lines, the macrophages were more sensitive to AgNP treatment than MCF-7 cancer cells. The stimulation of cancerous and non-cancerous cells generated the production of radical oxygen species (ROS), that could lead to oxidative stress and damaged cell membranes resulting in LDH leakage. Although our previously published results showed the high antimicrobial potential of AgNPs from strain SH11, their mutagenic effect in *S. typhimurium* cells was not found in the present study, which suggests the different mechanism of action in bacterial cells. As synthesized AgNPs display a high cytotoxic effect against cancer cells, their further functionalization by modifications of capping agents could lead to their use in targeted cancer therapy without side effects against healthy cells. Based on present and previously published studies, it is concluded that AgNPs biosynthesized from actinobacterial strain SH11 have a high potential in biomedicine.

## Figures and Tables

**Table 1:** Values of IC_50_ obtained by MTT and LDH assay.

**MTT Assay**	**LDH Assay**
IC_50_ (µg·mL^−1^)	IC_50_ (µg·mL^−1^)
**RAW 264.7 cells**	
3.78 ± 0.02	43.5 ± 0.08
**MCF-7 cells**	
4.78 ± 0.03	112.9 ± 0.08

**Table 2:** Genotoxicity of silver nanoparticles in *Salmonella typhimurium* TA98 test strain.

Dose (µg/plate)	Number of Bacterial Colonies/Plate (Mean ± SD)
negative control	20.0 ± 4
0.15	24.0 ± 1
0.25	18.0 ± 3
0.5	10.0 ± 1
1.0	4.0 ± 2
1.5	2.0 ± 2
3.0	4.0 ± 1
6.0	T
12.5	T
25.0	T
37.5	T
50.0	T
100.0	T
positive control	1084 ± 35

Key: SD represents standard deviation; T denotes toxicity detected at this and higher doses as a reduction in the frequency of spontaneous mutations.

**Table 3:** Identification of proteins associated with silver nanoparticles synthesized from actinobacterial strain SH11.

Band Number	Accession	Description	Taxonomy	Coverage (%)	MW (kDa)	Calculated pI	Score
**01**	WP_108997678.1	Hypothetical protein, partial	*Escherichia coli*	43.0	44.0	8.47	4614
	WP_040269305.1	OprD family porin	*Pseudomonas rhodesiae*	43.0	unknown	5.73	1759
	WP_094066152.1	MULTISPECIES: OprD family porin	*Pseudomonas fluorescens* group	49.0	unknown	5.73	1650
	WP_016712004.1	MULTISPECIES: OprD family porin	*Pseudomonas*	43.0	unknown	4.91	1445
**02**	WP_108997678.1	Hypothetical protein, partial	*Escherichia coli*	42.0	38.0	8.47	4376
	WP_008643071.1	MULTISPECIES: porin	*Cupriavidus*	44.0	unknown	8.81	2241
	WP_008645123.1	Porin	*Cupriavidus* sp. HMR-1	54.0	unknown	9.08	1951
	WP_103519360.1	Porin	*Ralstonia pickettii*	52.0	unknown	9.44	1477

Key: pI; isoelectric point.
